# Temperature-Dependent Thumb Domain Dynamics of Xylanase TsaGH11: Insights from Molecular Dynamics Simulations

**DOI:** 10.3390/ijms27156869

**Published:** 2026-07-31

**Authors:** Ki Hyun Nam

**Affiliations:** College of General Education, Kookmin University, Seoul 02707, Republic of Korea; structure@kookmin.ac.kr

**Keywords:** GH11 xylanase, temperature-dependent flexibility, thumb domain, substrate-binding cleft, molecular dynamics simulation

## Abstract

Xylanases catalyze the hydrolysis of β-1,4-xylosidic linkages in xylan, a major component of plant cell walls, and are widely used in the food, feed, pulp and paper, and biofuel industries. GH11 xylanase from the hemicellulose-degrading bacterium *Thermoanaerobacterium saccharolyticum* (TsaGH11) exhibits high catalytic activity, making it an attractive enzyme for industrial applications. The flexibility of the thumb domain of TsaGH11 has been investigated under cryogenic and room temperature conditions; however, the substrate recognition mechanism of TsaGH11 at the optimal temperature is unknown. To better understand the molecular mechanism of substrate recognition, the high-resolution crystal structure of TsaGH11 was determined at 1.4 Å resolution. All-atom molecular dynamics simulations at 300, 320, 340, and 360 K revealed that increasing the temperature induced fluctuations in the substrate-recognizing thumb domain. At an optimal temperature of 340 K, the substrate-binding cleft of TsaGH11 predominantly adopted a closed conformation. However, the thumb domain exhibited larger fluctuations at 340 K than at other temperatures, sampling both open and closed conformations, suggesting that substrate recognition in TsaGH11 proceeds through a conformational selection-like mechanism. At 360 K, TsaGH11 unfolded partially at a site opposite the substrate-binding cleft, providing potential targets for protein engineering to improve its thermostability for industrial applications. These findings provide a better understanding of the molecular mechanism of TsaGH11 and offer valuable guidance for the rational engineering of GH11 xylanases for industrial applications.

## 1. Introduction

Lignocellulosic biomass is the most abundant renewable carbon resource on Earth and plays a central role in the development of sustainable biobased industries [1,2]. Xylan constitutes a significant fraction of lignocellulosic biomass, and its efficient degradation is essential for the conversion of renewable biomass into value-added products [3,4]. Xylanases are glycoside hydrolases (GHs) that catalyze the hydrolysis of β-1,4-xylosidic linkages in xylan, the major hemicellulosic component of plant cell walls [5,6]. Xylanases have been widely utilized in a broad range of industrial applications [4,7]. In the pulp and paper industry, xylanases are used for biobleaching to reduce the need for harsh chemical treatments [8,9]. In the food and feed industries, xylanases improve dough handling, bread quality, and nutrient availability by degrading nonstarch polysaccharides [10,11]. Xylanases catalyze the hydrolysis of xylan into xylose and xylo-oligosaccharides, facilitating the enzymatic saccharification of lignocellulosic biomass into fermentable sugars that can be further converted into biofuels and value-added biochemicals in sustainable bioprocessing [12,13,14].

Based on sequence similarity and structural features, xylanases are classified into several GH families [15,16], of which GH10 and GH11 are the most extensively studied [17,18]. GH11 xylanases enable high catalytic efficiency and substrate specificity toward xylan backbones [19]. To obtain efficient xylanases with industrial applicability, xylanases are continuously being discovered from novel microbial strains or further improved through protein engineering [20]. Crystal structures of GH11 xylanases from various species have provided valuable insights into their overall fold, substrate-recognition features, and catalytic mechanisms, thereby advancing our understanding of enzymatic function and improving protein engineering [19,21,22]. The thumb domain located above the substrate-binding site of GH11 xylanase plays a crucial role in substrate binding [23,24]. During GH11 catalysis, the open conformation of the thumb domain is considered favorable for substrate accessibility [23,25]. Upon substrate binding, the thumb domain partially closes, forming an intermediate state through an induced-fit motion [24,26,27]. Therefore, the conformation of the thumb domain determines the geometry and volume of the substrate-binding cleft and provides a molecular basis for substrate recognition.

GH11 xylanase from the hemicellulose-degrading thermophilic bacterium *Thermoanaerobacterium saccharolyticum* (TsaGH11) exhibits high catalytic efficiency toward xylan, with maximal activity at 70 °C, suggesting its potential in industrial applications [28]. The crystal structure of TsaGH11 collected at cryogenic temperature exhibited a clear electron density map corresponding to the solvent-exposed thumb domain [28,29], indicating a rigid conformation of the thumb domain. By contrast, in the room-temperature structure of TsaGH11 determined using serial synchrotron crystallography, the electron density corresponding to the solvent-exposed thumb domain of TsaGH11 was disordered [20], indicating that increased temperature induces enhanced flexibility of the thumb domain without a single preferred conformation.

Understanding the conformation of the thumb domain of GH11 xylanase is essential for elucidating the molecular mechanism of substrate recognition [19,23,26]. Temperature is critical for enzymatic activity and protein structure, including molecular flexibility [30,31], which can influence the conformation of xylanase, including the thumb domain. For example, room-temperature structures of GH11 xylanases from *T. saccharolyticum* and *Trichoderma longibrachiatum* revealed increased molecular flexibility in substrate recognition regions and differences in hydration environments compared with structures collected at cryogenic temperatures [28,32].

To identify the substrate recognition mechanism, structural information at the optimal temperature for enzymatic activity is essential. However, structure determination of TsaGH11 at its maximal enzymatic activity (~70 °C) is challenging because crystal packing is thermally damaged. Consequently, it is unclear whether the thumb domain of TsaGH11 at its optimal enzymatic temperature adopts specific preferred open conformations that facilitate efficient substrate binding or exhibits enhanced flexibility similar to that observed in the room-temperature structure, both of which are critical for understanding the substrate recognition mechanism.

To better understand the substrate binding mechanism of TsaGH11 at its optimal enzymatic temperature, all-atom molecular dynamics (MD) simulations were performed using a high-resolution crystal structure of 1.4 Å resolution that was determined in this study. Molecular fluctuations of TsaGH11 were analyzed using two initial model structures with open and closed conformations. Temperature-dependent conformational changes in the substrate-binding cleft were analyzed. The unfolding regions of MD structures at high temperatures were analyzed to provide insights for engineering proteins with improved thermostability. These studies provide insights into the structural dynamics and substrate recognition of TsaGH11 and offer a molecular basis for the rational engineering of GH11 xylanases with improved efficiency in industrial applications.

## 2. Results

### 2.1. Overall Structure of TsaGH11

To utilize the accurate structural model for MD simulation, the high-resolution crystal structure of TsaGH11 was determined at 1.4 Å resolution, with final R_work_ and R_free_ values of 0.1748 and 0.1884, respectively (Table 1).

**Figure 1:**
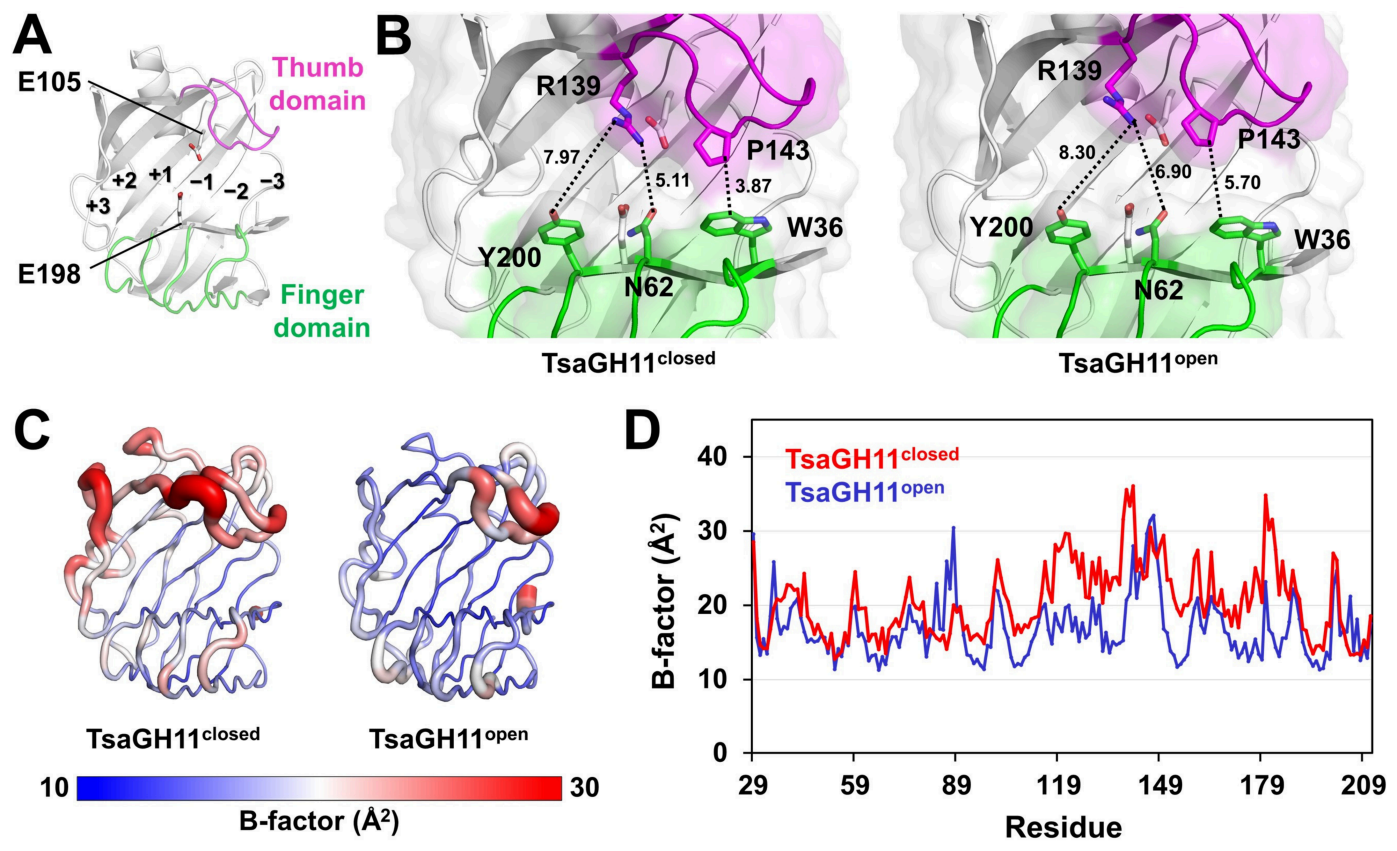
High-resolution crystal structure of TsaGH11. (**A**) Cartoon representation of TsaGH11. (**B**) Surface structure of the closed and open conformations of TsaGH11. (**C**) B-factor putty analysis of the closed and open conformations of TsaGH11. (**D**) B-factor plot of the closed (red) and open (blue) conformations of TsaGH11.

The overall structure of TsaGH11 was highly similar to reported TsaGH11 structures [28,29,33]. Briefly, TsaGH11 adopted a typical β-jelly roll fold characteristic of GH11 xylanases and contained six subsites (from −3 to +3), forming the thumb, finger, and palm domains (Figure 1A). The catalytic residues Glu105 (nucleophile) and Glu198 (proton donor) were in the central region of the substrate-binding cleft between subsites −1 and +1 (Figure 1A). Two TsaGH11 molecules were present in the asymmetric unit, exhibiting distinct closed and open conformations of the substrate-binding cleft, designated as TsaGH11^closed^ and TsaGH11^open^, respectively (Figure 1B), due to crystal packing effects [28,29]. In the crystal lattice, TsaGH11^open^ participated extensively in crystal packing interactions with neighboring TsaGH11 molecules, whereas TsaGH11^closed^, including the thumb domain, was exposed to solvent regions within the crystal lattice. The closest distance between the catalytic residues Glu105 and Glu198 in TsaGH11^closed^ and TsaGH11^open^ was 5.27 and 5.20 Å, respectively. The closest distance between Trp36 in the finger domain and Pro143 in the thumb domain differed significantly between the two conformations—3.87 and 5.70 Å in TsaGH11^closed^ and TsaGH11^open^, respectively (Figure 1B). The closest distances between Asn62 in the finger domain and Arg139 in the thumb domain differed significantly between the two conformations—5.11 and 6.90 Å in TsaGH11^closed^ and TsaGH11^open^, respectively (Figure 1B).

B-factor analysis revealed notable differences in the distribution of flexible regions between the two conformations (Figure 1C,D). In TsaGH11^closed^, regions outside the β-jelly roll fold core, mainly comprising loop regions, exhibited relatively higher B-factors, whereas in TsaGH11^open^, high flexibility was primarily observed in the thumb domain and loop regions between β-strands. Average B-factor values of TsaGH11^closed^ and TsaGH11^open^ were approximately 20.45 and 16.99 Å^2^, respectively. These results indicate that crystal packing can significantly influence local flexibility in the crystal structure, potentially leading to differences from the intrinsic conformational dynamics of the protein in solution. Based on crystal packing effects and structural analysis, the closed conformation of TsaGH11 was considered more biologically relevant for molecular flexibility because it was more exposed to solvent regions in the crystal lattice.

### 2.2. MD Simulation of TsaGH11

The molecular flexibility observed in MD simulations can be influenced by the choice of the initial structural model [34,35]. Therefore, the simulation outcomes are expected to differ between the open and closed conformations of TsaGH11. Although the closed conformation of TsaGH11 is considered more biologically relevant, particularly with respect to crystal packing effects, in this study, MD simulations were conducted for both conformations to obtain a more comprehensive understanding of the molecular flexibility of TsaGH11 and to assess the extent to which the initial structural model influences conformational dynamics. In a previous study, the relative enzymatic activities of TsaGH11 at 30, 50, 70, and 90 °C were approximately 55%, 90%, 100%, and 30%, respectively [28]. Based on this temperature-dependent enzyme activity profile, MD simulations were performed at 300, 320, 340, and 360 K for 500 ns to investigate temperature-dependent structural changes in TsaGH11.

#### 2.2.1. Temperature-Dependent MD Simulation of TsaGH11^closed^

**Figure 2:**
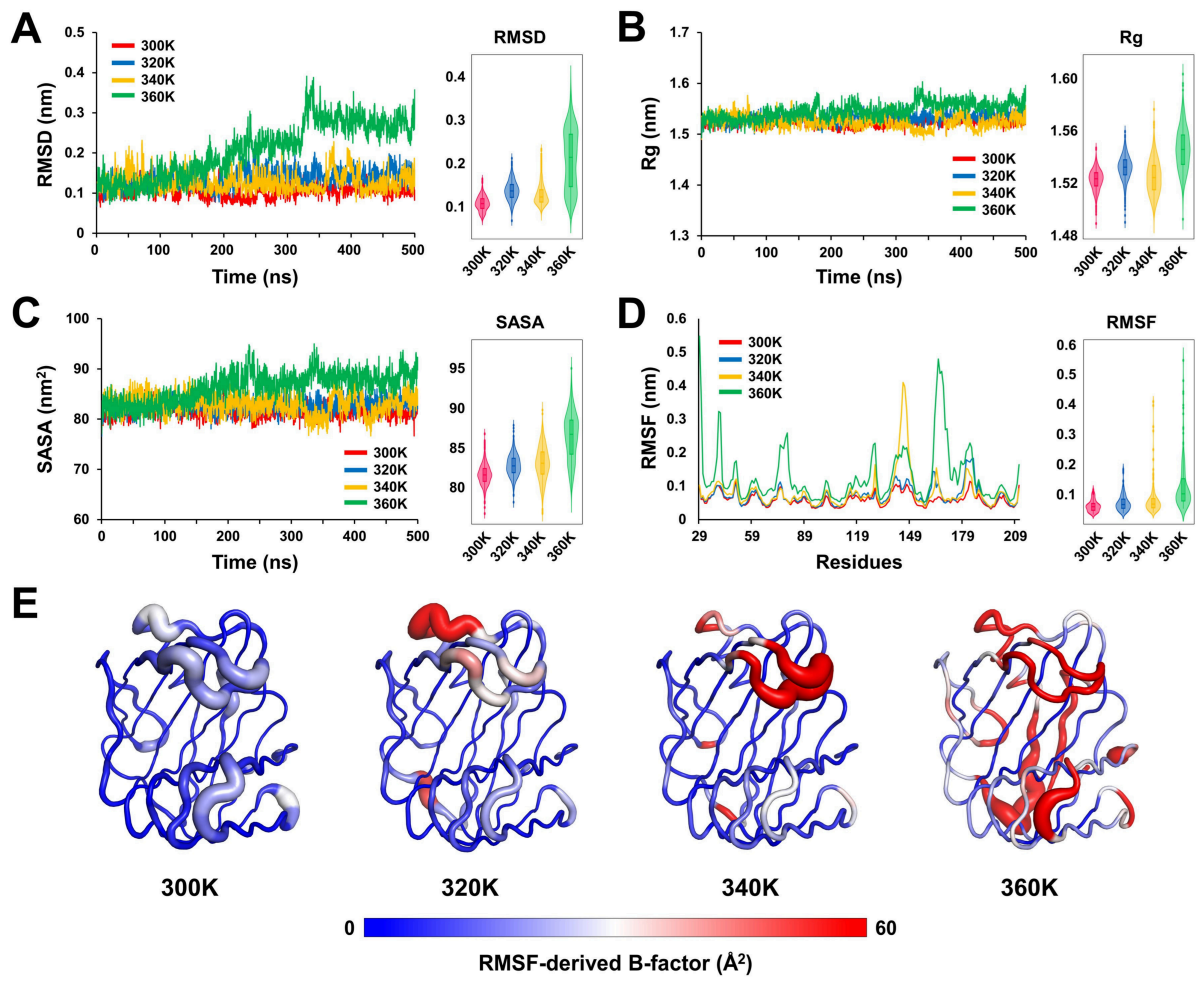
MD simulation of TsaGH11^closed^. MD analysis for (**A**) RMSD, (**B**) Rg, (**C**) SASA, and (**D**) RMSF. (**E**) Average backbone B-factor profiles calculated from MD trajectories of TsaGH11^closed^ at 300, 320, 340, and 360 K based on the RMSF value.

The RMSD values of TsaGH11^closed^ at 300 K fluctuated narrowly at 0.07–0.10 nm, indicating a highly stable conformation (Figure 2A). At 320 and 340 K, RMSD values increased modestly relative to those at 300 K, typically ranging from ~0.09–0.14 nm, with slightly broader fluctuations, suggesting enhanced flexibility without loss of global structural stability (Figure 2A). By contrast, at 360 K, RMSD behaved significantly differently (Figure 2A). After an initial equilibration phase, RMSD increased progressively over time and reached values of ~0.25–0.35 nm, with several significant spikes observed during the mid-to-late stages of simulation, indicating significant large-scale conformational rearrangements (Figure 2A). Average RMSD values of TsaGH11^closed^ at 300, 320, 340, and 360 K were 0.106, 0.136, 0.126, and 0.208 nm, respectively, indicating a significant structural change at 360 K.

The Rg values of TsaGH11^closed^ at 300, 320, and 340 K exhibited similar fluctuations at ~1.51–1.53 nm without any significant systematic drift, indicating that the protein maintains an overall compact structure (Figure 2B). At 360 K, the Rg value showed a slight upward shift with a broader distribution, reaching a maximum of ~1.60 nm, indicating partial loosening of global packing (Figure 2B).

SASA values at 300, 320, and 340 K were within a similar range, whereas a slight upward drift was observed at 360 K (Figure 2C). Average SASA values of TsaGH11^closed^ at 300, 320, 340, and 360 K were 81.56, 82.72, 83.02, and 86.35 nm^2^, respectively.

RMSF analysis showed that most residues of TsaGH11^closed^ at 300 K exhibited RMSF values of <0.20 nm, indicating a relatively rigid structure (Figure 2D). Compared to RMSF values at 300 K, RMSF values at 320 K increased moderately in an α-helix region (Lys180–Leu186) on the opposite side of the substrate-binding cleft (Figure 2D); however, these fluctuations were modest relative to those observed at higher temperatures. At 340 K, significant fluctuations were observed in the thumb domain (Pro143–Gly147), with RMSF values reaching ~0.40 nm, indicating enhanced flexibility of the thumb domain near the optimal enzymatic activity temperature (Figure 2D). At 360 K, significant fluctuations were observed across the N-terminal (Thr29–Tyr32) and opposite sides of the substrate-binding cleft (Pro74–Tyr80 and Arg162–Leu186), with RMSF values ranging from ~0.45 to 0.55 nm (Figure 2D), whereas the fluctuation of the thumb domain decreased compared with that at 340 K.

RMSF-derived B-factor putty representation showed that at 300 and 320 K, TsaGH11 exhibited relatively rigid fluctuations at the substrate-binding cleft and active site (Figure 2E). At 340 K, the thumb domain exhibited significantly increased flexibility (Figure 2E), indicating that the conformation of the substrate-binding cleft becomes more variable. By contrast, at 360 K, the distribution of highly fluctuating regions expanded to include not only the substrate-binding region but also the core region of the β-jelly roll fold (Figure 2E).

#### 2.2.2. Temperature-Dependent MD Simulation of TsaGH11^open^

**Figure 3:**
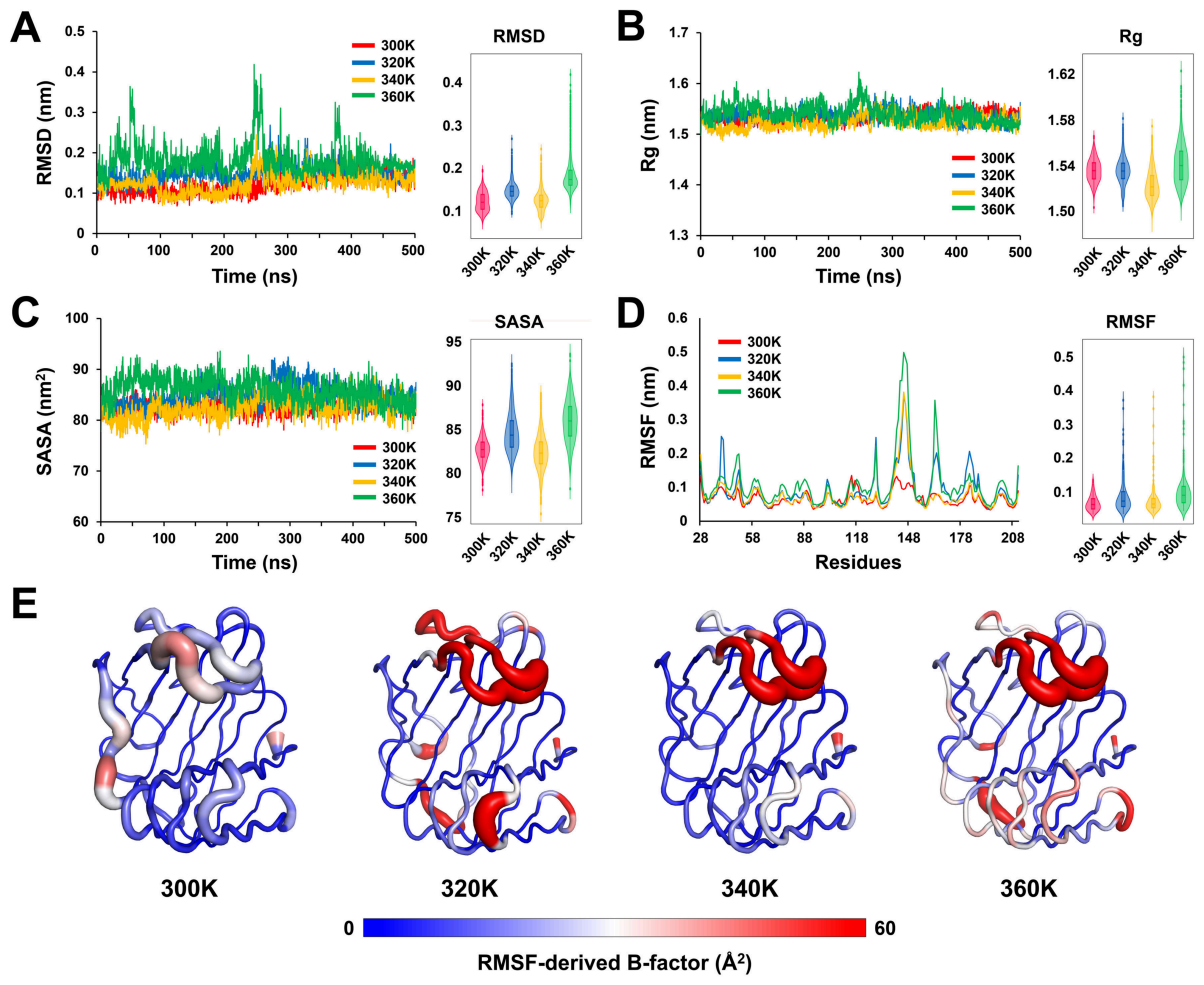
MD simulation of TsaGH11^open^. (**A**) RMSD, (**B**) Rg, (**C**) SASA, and (**D**) RMSF. (**E**) Average backbone B-factor profiles calculated from molecular dynamics trajectories of TsaGH11^open^ at 300, 320, 340, and 360 K based on the RMSF value.

RMSD analyses showed that at 300 K, TsaGH11^open^ exhibited the lowest RMSD values, ranging from ~0.08 to 0.13 nm, with minimal fluctuations, indicating a highly stable conformation (Figure 3A). At 320 K, RMSD values increased modestly relative to those at 300 K, typically ranging from ~0.11 to 0.18 nm (Figure 3A). Despite increased thermal motion, the RMSD profile was stable without long-term drift, suggesting enhanced flexibility while preserving overall fold. At 340 K, the RMSD distribution became broader (Figure 3A). Transient increases in RMSD were observed around the mid-simulation region (240–250 ns); however, these deviations were reversible, and RMSD subsequently returned to a stable baseline (Figure 3A), indicating local conformational rearrangements. At 360 K, RMSD values exhibited the largest fluctuations, ranging from ~0.10 to 0.40 nm (Figure 3A). Although no continuous upward drift was observed, fluctuations were higher than those at other temperatures, even after stabilization beyond 450 ns (Figure 3A). Average RMSD values at 300, 320, 340, and 360 K were 0.1213, 0.1475, 0.1240, and 0.1857 nm, respectively.

The Rg values of TsaGH11 at 300, 320, and 340 K were stable at 1.49–1.58 nm (Figure 3B). At 360 K, Rg values were initially slightly higher (1.52–1.62 nm) than those at lower temperatures; however, after ~400 ns, Rg converged to 1.50–1.57 nm, comparable to those at other temperatures (Figure 3B).

Average SASA values at 300, 320, 340, and 360 K were 82.68, 84.64, 82.33, and 85.93 nm^2^, respectively (Figure 3C). No significant changes were observed in temperature-dependent SASA trends, indicating the absence of persistent expansion of the solvent-accessible surface upon temperature increase.

RMSF analysis at 300 K revealed low RMSF values (less than ~0.14 nm), indicating that the thumb, finger, and palm domains maintained a rigid fold (Figure 3D). At 320 K, RMSF values increased moderately across loop regions and the thumb domain compared with those at 300 K; however, the N-terminal loop region (Val40–Gly41) in the finger domain exhibited relatively higher flexibility than at other temperature conditions. At 340 K, the overall fluctuation pattern was similar to that at 300 K; however, the RMSF values of the thumb domain (Pro143–Gly147) increased markedly, reaching a maximum of ~0.38 nm, corresponding to up to a threefold increase compared with those observed at 300 K. At 360 K, the RMSF peaks increased significantly in the thumb domain, reaching ~0.50 nm, and loop regions (Arg162–Gly165), reaching ~0.35 nm, located on the opposite side of the substrate-binding pocket (Figure 3D). Despite these significant fluctuations, the core β-jelly roll fold maintained low RMSF values, indicating that increased flexibility was localized and did not affect overall structure.

RMSF-derived B-factor putty representation showed that at 300 K, TsaGH11^open^ exhibited relatively rigid fluctuations at the substrate-binding cleft and active site (Figure 3E). At 340 K, the thumb domain exhibited significantly increased flexibility, whereas other regions were relatively rigid (Figure 3E). By contrast, at 320 and 360 K, not only the thumb domain but also several loop regions showed partially increased fluctuations, indicating a more distributed flexibility across the structure (Figure 3E).

### 2.3. Multiple Conformations of the Thumb Domain in TsaGH11 near the Optimal Temperature

The initial model structure critically influences MD simulation results [34,35]. Consistently, this effect was observed in this study when comparing MD simulations initiated from the open and closed conformations of TsaGH11. Compared to TsaGH11^open^, TsaGH11^closed^ is considered to be more representative of its intrinsic molecular flexibility because it is less affected by interactions with neighboring molecules in the crystal packing lattice. To better understand temperature-dependent structural changes, trajectory snapshots and representative MD conformations of TsaGH11^closed^ were analyzed.

**Figure 4:**
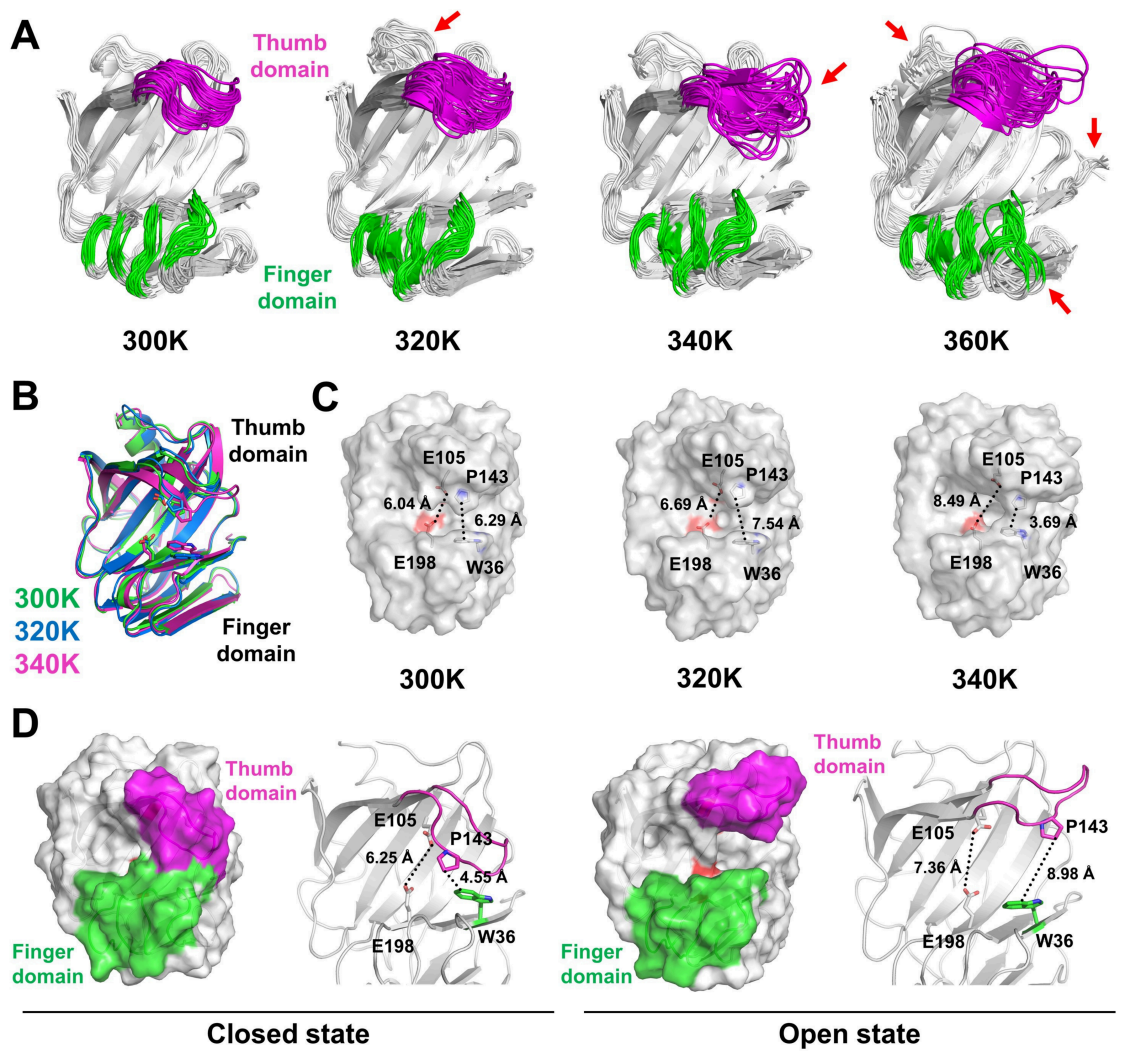
Temperature-dependent conformational dynamics of TsaGH11^closed^ revealed by MD simulations. (**A**) Structural ensembles of TsaGH11^closed^ extracted from MD trajectories at 300, 320, 340, and 360 K. Regions exhibiting relatively high fluctuations and positional shifts are indicated by red arrows. (**B**) Superimposition of representative MD structures of TsaGH11^closed^ at 300 K (green), 320 K (blue), and 340 K (magenta). (**C**) Analysis of the distances between catalytic residues and widths of the substrate-binding cleft of TsaGH11^closed^ at 300, 320, and 340 K. (**D**) Extracted structures representing the maximally closed and open conformations between the thumb and finger domains from the MD trajectories of TsaGH11^closed^ at 340 K.

Analysis of structural ensembles at different temperatures showed that the overall fold of TsaGH11 was well preserved from 300 to 360 K, with the β-jelly roll fold remaining structurally stable (Figure 4A). However, significant differences in local fluctuations were observed in the thumb and finger domains. At 300 K, TsaGH11 exhibited the lowest overall structural fluctuation compared with other temperature models, with only moderate flexibility observed in the finger and thumb domains. At 320 K, the position shifts and fluctuations in the thumb and finger domains were slightly increased (Figure 4A). Increased fluctuations were observed in the loop regions on the opposite side of the substrate-binding cleft, showing higher flexibility than that in models at other temperatures. At 340 K, the thumb domain exhibited the largest positional shift and fluctuations among all temperature models (Figure 4A). At 360 K, the thumb and finger domains as well as the N-terminus exhibited high positional shifts and fluctuations (Figure 4A). In these regions, the positional shift in the thumb domain at 360 K was reduced compared with that at 340 K. Overall, these results indicate that temperature increase induces local fluctuations in the thumb and finger domains as well as loop regions opposite the substrate-binding site and the N-terminus.

Representative structure analysis enables summarizing dominant conformational states sampled during MD simulation, providing major features of the conformational ensemble for downstream analyses such as functional interpretation or docking [36,37]. Clustering analysis revealed that the MD trajectories of TsaGH11 at 300, 320, and 340 K predominantly formed a single major cluster, whereas the MD trajectory at 360 K exhibited multiple clusters. MD trajectory and secondary structure analyses indicated that partial unfolding occurred in the simulations at 360 K (see below). Therefore, the representative MD structure at 360 K was excluded from comparative structural analyses.

Superposition of representative MD structures of TsaGH11^closed^ at 300, 320, and 340 K revealed distinct positional differences in the thumb and finger domains (Figure 4B), indicating that temperature affects the conformation of the substrate-binding cleft and active-site region. The distances between the two catalytic residues (Glu105 and Glu198) of TsaGH11 at 300, 320, and 340 K were 6.04, 6.69, and 8.49 Å, respectively (Figure 4C). These results indicate that the distance between the catalytic residues becomes larger near the optimal temperature for enzyme activity. The time-series analysis of the Glu105–Glu198 distance demonstrated temperature-dependent changes in the catalytic-site geometry of TsaGH11 (Supplementary Figure S1). The distance remained relatively stable at 300 K, with an average value of 6.83 ± 0.68 Å. At 320 K and 340 K, the average distance increased slightly to 7.77 ± 0.89 Å and 7.39 ± 0.87 Å, respectively. The greatest conformational variability occurred at 360 K, where the average distance increased to 8.09 ± 1.19 Å. The time-series analysis showed that, rather than exhibiting a continuous increase in distance, the Glu105–Glu198 pair repeatedly underwent distance expansion and contraction, with the amplitude of these fluctuations progressively increasing as the temperature increased (Supplementary Figure S1).

Meanwhile, the distances between Pro143 in the thumb domain and Trp36 in the finger domain were 6.29, 7.54, and 3.69 Å at 300, 320, and 340 K, respectively (Figure 4C). These structural analyses revealed a clear temperature-dependent trend in the distance changes between catalytic residues as well as between the thumb and finger domains. The time-series analysis of the Trp36–Pro143 distance, which reflects the opening of the substrate-binding cleft, revealed temperature-dependent conformational changes (Supplementary Figure S2). At 300 K and 320 K, the thumb region predominantly remained in a relatively open conformation, with average distances of 6.40 ± 1.28 Å and 7.42 ± 1.34 Å, respectively. At 340 K, the average distance decreased slightly to 5.91 ± 1.89 Å; however, the substantially larger fluctuations indicate frequent transitions between the open and closed conformations. At 360 K, the average distance increased markedly to 8.24 ± 2.34 Å, accompanied by the largest fluctuations among all temperatures, indicating frequent sampling of highly open conformations. The time-series trajectories at 300, 320, and 340 K showed repeated transitions between shorter and longer Trp36–Pro143 distances within a relatively constant range throughout the simulations (Supplementary Figure S2). In contrast, at 360 K, the Trp36–Pro143 distance remained predominantly in the open conformation after approximately 320 ns, suggesting stabilization of the open state during the latter half of the simulation (Supplementary Figure S2).

To further quantify the conformational states of the thumb region, the Trp36-Pro143 distance was classified into open (>0.5 nm) and closed (≤0.5 nm) conformations. The cutoff value of 5 Å was selected based on the experimentally determined open and closed crystal structures (Table 2). Using this criterion, the open conformation accounted for 82.69% of the simulation at 300 K, increased to 94.65% at 320 K, decreased to 59.00% at 340 K because of frequent transitions between the open and closed states, and increased again to 91.19% at 360 K. Correspondingly, the closed conformation occupied 17.31%, 5.35%, 41.00%, and 8.81% of the simulations at 300, 320, 340, and 360 K, respectively. These results indicate MD models at 340 K exhibit the greatest interchange between the open and closed conformations.

To understand the maximum fluctuation of the thumb domain of TsaGH11 at the optimal enzymatic temperature, the most closed and most open conformations were extracted from the trajectory of the TsaGH11^closed^ simulation at 340 K and compared. Surface structure analysis revealed that in the closed state of TsaGH11^closed^, the substrate-binding cleft is shielded by the closure of the thumb and finger domains, whereas in the open state of TsaGH11^closed^, the entire substrate-binding cleft is fully exposed (Figure 4D). In the closed state of the thumb domain of TsaGH11^closed^, the distance between Pro143 in the thumb domain and Trp36 in the finger domain was 4.55 Å, and the distance between Glu105 and Glu198 was 6.25 Å (Figure 4D). In the open state of the thumb domain of TsaGH11^closed^, the distance between Trp36 and Pro143 increased to ~8.98 Å, and the distance between Glu105 and Glu198 increased to 7.36 Å (Figure 4D). Collectively, these results indicate that at optimal enzymatic temperature, the large fluctuations of the thumb domain enable TsaGH11^closed^ to adopt both open and closed conformations of the substrate-binding cleft.

**Figure 5:**
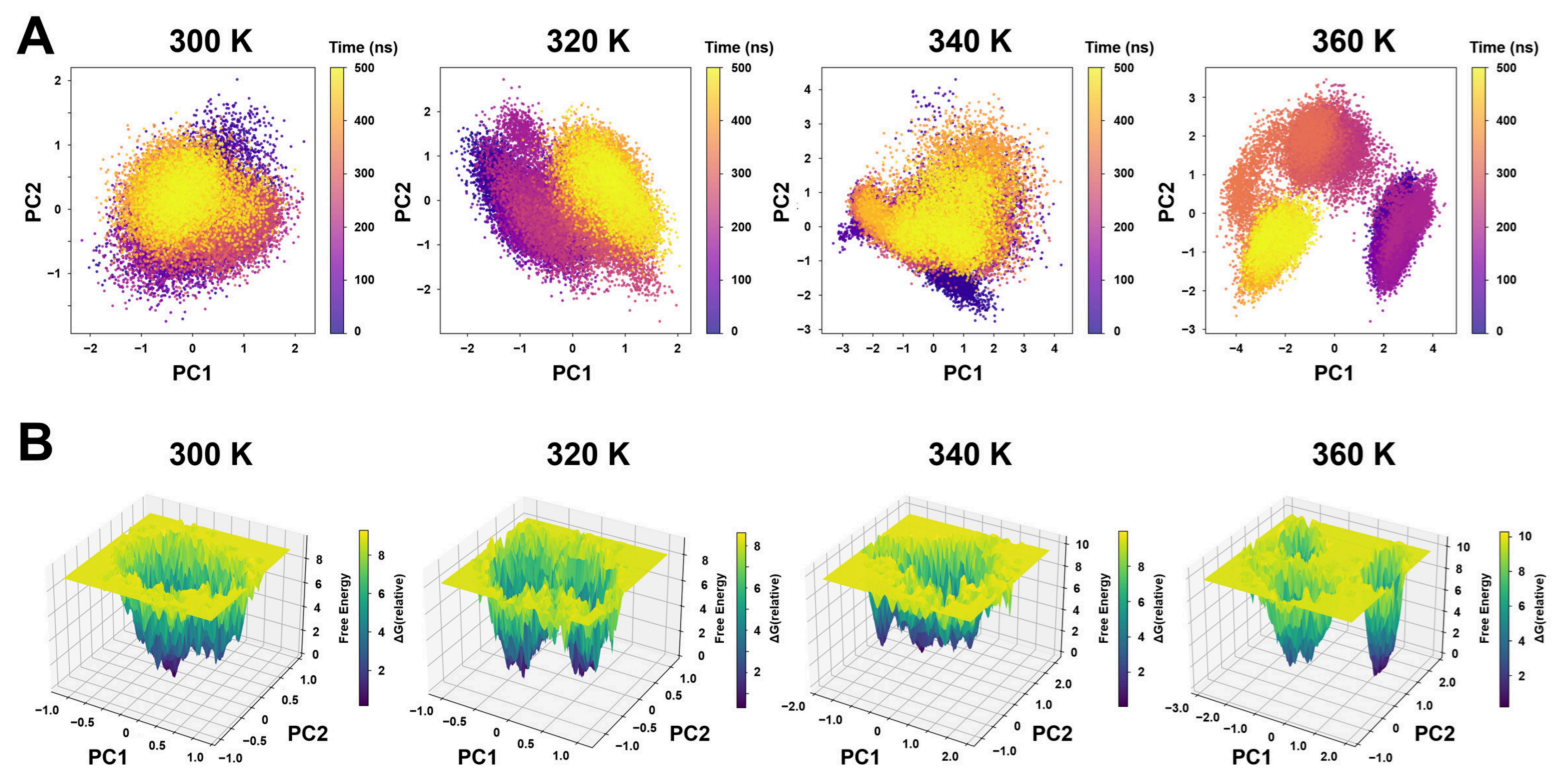
Principal component analysis (PCA) and free energy landscape (FEL) analyses of TsaGH11^close^ at different temperatures. (**A**) PCA projections of MD trajectories at 300, 320, 340, and 360 K. Colors represent simulation time. (**B**) FEL generated from the first two principal components at 300, 320, 340, and 360 K.

To better understand the temperature-dependent conformational dynamics of TsaGH11, principal component analysis (PCA) and free energy landscape (FEL) analyses were performed using the MD trajectories at 300, 320, 340, and 360 K. PCA was performed to characterize the conformational space sampled by TsaGH11 during the MD simulations (Figure 5A). At 300 K, the conformational ensemble occupied a relatively compact region in the PC1–PC2 space. At 320 K, the conformational distribution became broader and was partially separated into two major regions (Figure 5A). At 340 K, the conformational ensemble sampled a wider region of the PC1–PC2 space than at lower temperatures. At 360 K, the conformational ensemble was distributed into several distinct clusters, indicating increased conformational sampling (Figure 5A).

The FELs constructed from the first two principal components showed that, at 300 K, a single dominant energy basin was observed (Figure 5B). At 320 K, two major energy basins became apparent (Figure 5B). At 340 K, the free energy landscape became broader with multiple shallow energy minima (Figure 5B). At 360 K, several distinct low-energy basins were observed, indicating the presence of multiple stable conformational states during the simulation (Figure 5B).

Overall, PCA and FEL analyses showed that TsaGH11 exhibited distinct conformational dynamics at each temperature rather than a progressive temperature-dependent trend.

### 2.4. Analysis of the Thermal Unfolding Region of TsaGH11

**Figure 6:**
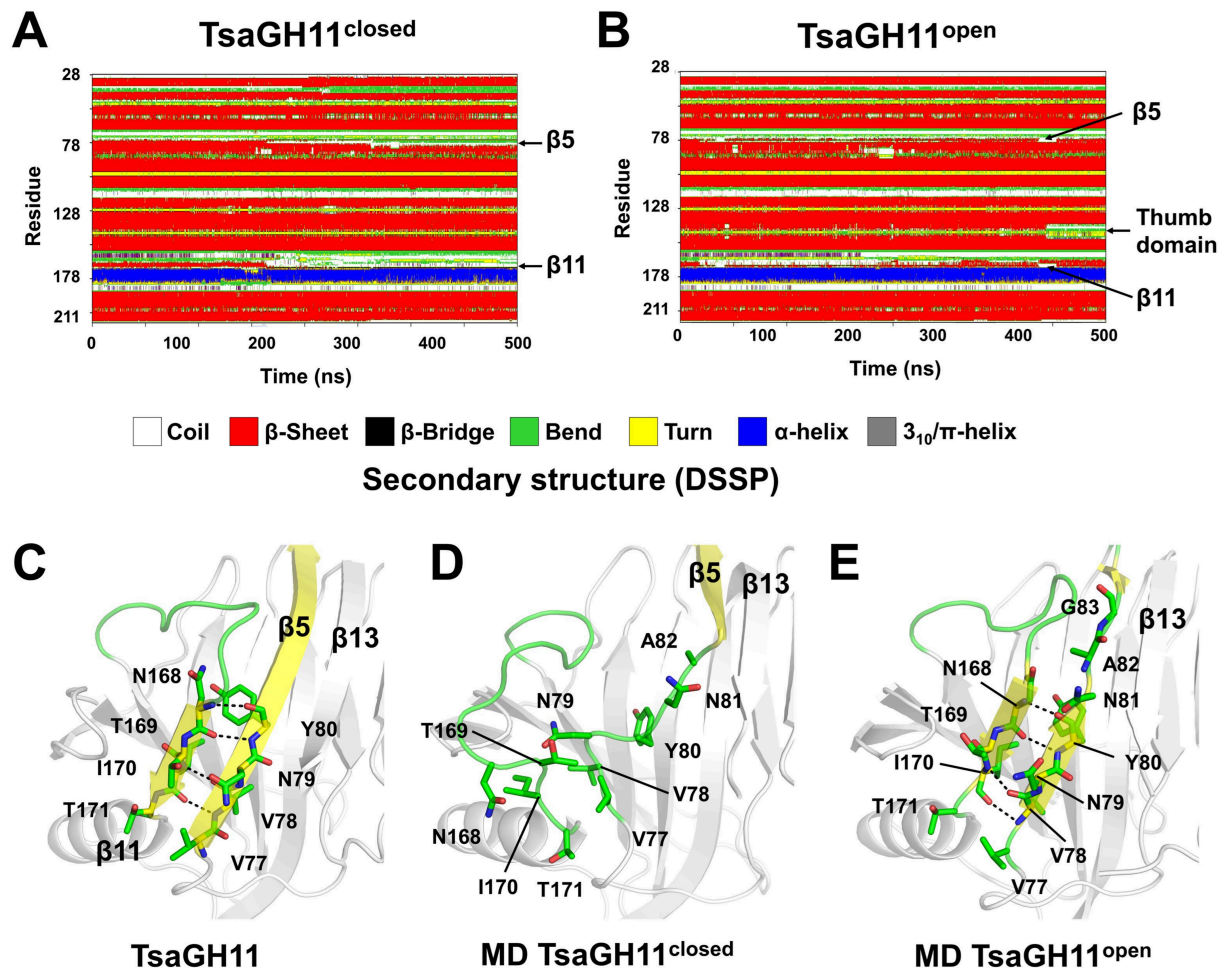
Analysis of the secondary structures and thermal unfolding regions of TsaGH11 at 360 K using MD simulations. Time evolution of the secondary structures of (**A**) TsaGH11^closed^ and (**B**) TsaGH11^open^ at 360 K over the 500 ns MD production trajectories. (**C**) Crystal structure of TsaGH11^closed^ exhibits a stable β-sheet formed between strands β5 and β11. Final snapshots (500 ns) of the MD trajectories of (**D**) TsaGH11^closed^ and (**E**) TsaGH11^open^ obtained at 360 K exhibit thermal unfolding in the β-sheet regions.

Analysis of thermal unfolding mechanisms at high temperatures using MD simulations can provide insights into rational design strategies for enhancing protein thermostability [38,39]. RMSD, Rg, SASA, and RMSF analyses showed that TsaGH11^closed^ and TsaGH11^open^ maintained the β-jelly roll fold across all temperatures. However, at 360 K, TsaGH11^closed^ exhibited high fluctuations without a clear temperature-dependent trend, which may indicate partial unfolding induced by increased temperature. To determine thermally sensitive regions that affect protein unfolding, the time evolution of the secondary structure was analyzed. For TsaGH11 at 300, 320, and 340 K, the core secondary structures, including β-strands and α-helices, were preserved throughout the simulations (Supplementary Figure S3). By contrast, the core secondary structure was partially unfolded at 360 K. In TsaGH11^closed^, strands β5 and β11, on the opposite side of the substrate-binding cleft within the finger domain, exhibited a β-strand-to-coil transition at ~215 ns (Figure 6A). This transition persisted for the remainder of the simulation, indicating an irreversible local unfolding event in these regions. TsaGH11^open^ at 360 K exhibited a similar β-strand-to-coil transition at the β5 (Val77–Asn88) and β11 (Asn168–Thr171) regions during the simulation (Figure 6B). However, after ~450 ns, these regions partially reformed β-strand conformations, indicating a reversible secondary structure transition (Figure 6B). After ~430 ns, a β-strand-to-coil transition was observed in regions surrounding the thumb domain (Figure 6B); however, the final snapshot from the trajectory showed reversibility, with these regions undergoing a coil-to-β-strand transition.

To further understand the regions responsible for structural unfolding at increased temperatures, the crystal structure of TsaGH11 in its stably folded state was compared with the final snapshot (500 ns) of the MD trajectories of TsaGH11^closed^ and TsaGH11^open^ obtained at 360 K. In the crystal structure of TsaGH11, strands β5 and β11 formed a stable β-sheet through hydrogen bond interactions between Val77–Tyr80 in β5 and Asn168–Thr171 in β11 (Figure 6C). By contrast, in TsaGH11^closed^, Val77–Ala82 in β5 and Asn168–Thr171 in β11 adopted coil conformations (Figure 6D), indicating the unfolding of the β-sheet fold involved in the protein architecture. In TsaGH11^open^, a stable β-sheet was maintained through main-chain hydrogen bonds between Val77–Tyr80 in β5 and Asn168–Thr171 in β11; however, Asn82 and Glu83 in β5 did not form hydrogen bond interactions with the neighboring β13 (Figure 6E). Overall, MD simulation results indicate that β5 and β11 of TsaGH11, on the opposite side of the substrate-binding site, are susceptible to temperature-induced unfolding.

## 3. Discussion

In this study, MD simulations were performed on two conformational states of TsaGH11—TsaGH11^closed^ and TsaGH11^open^. Both conformational states exhibited common structural features, including enhanced fluctuations in the thumb domain and thermally sensitive unfolding regions. However, results of detailed MD simulation analysis, such as RMSD and RMSF profiles, showed quantitative differences between the two systems. These findings indicate that MD simulation outcomes can vary depending on the initially selected structural model. Consistently, MD simulation outcomes were reportedly sensitive to the starting conformation used for trajectory generation [34,35]. The distinct MD simulation results obtained for TsaGH11^open^ and TsaGH11^closed^ provide an additional example illustrating that detailed MD can vary depending on the choice of the initial structural model.

The open conformation between the thumb and finger domains of GH11 xylanase widens the substrate-binding cleft and facilitates substrate accessibility [23,25]. An MD study of the thermophilic xylanase EvXyn11TS showed that the enzyme adopts a predominantly closed conformation at 310 K, whereas increased thumb-loop flexibility and a transition toward an open conformation occur at 340 K, suggesting enhanced substrate accessibility [40]. By contrast, comparing the representative MD structures of TsaGH11^closed^ at 300, 320, and 340 K revealed that increasing the temperature reduced the distance between the thumb and finger domains and increased the distance between the two catalytic glutamate residues. These distance changes suggest that temperature increase modulates the relative positioning of substrate recognition elements and catalytic residues. The open-to-closed conformational trend of the representative TsaGH11^closed^ upon increasing temperature observed in our MD simulations is distinct from those reported in other MD simulation studies of GH11 xylanases. These results suggest that, unlike canonical GH11 xylanases, TsaGH11 tends to favor a more closed substrate-binding cleft as the temperature approaches the optimal temperature for optimal catalytic activity. In addition to TsaGH11, temperature-dependent conformational changes in the substrate-binding cleft that differ from those of canonical xylanases have been reported in other xylanases. The mesophilic enzyme NpXyn11A maintained a relatively rigid thumb domain at 310 and 340 K due to a stabilizing salt bridge and exhibited a widened active-site cleft [40]. MD simulation analysis of the GH11 xylanase X11P and its engineered variant X11PNQ at 300, 350 and 400 K exhibited temperature-dependent differences in overall dynamic flexibility and hydrogen-bonding patterns, but no significant conformational shifts in the domains or temperature-dependent opening or closing of the substrate-binding cleft were observed [41]. Taken together, temperature-induced fluctuations and preferred conformational states of substrate-recognition domains vary among GH11 xylanases, indicating distinct and enzyme-specific strategies for achieving optimal catalytic activity.

The representative structure of TsaGH11 at 340 K exhibited a relatively closed conformation between the thumb and finger domains compared with those at 300 and 320 K. However, trajectory-based analyses revealed that at 340 K, the thumb domain sampled a broader range of open and closed conformations than at other temperatures. This observation indicates that at the optimal enzymatic temperature, the thumb domain does not adopt a single static conformation but undergoes continuous conformational fluctuations, dynamically transitioning between open and closed states. These results suggest that near the optimal enzymatic temperature, the substrate-binding cleft of TsaGH11 is predominantly biased toward a closed conformation, whereas the thumb domain can undergo large conformational changes. The transient adoption of an open conformation of the thumb domain may facilitate substrate access to the substrate-binding cleft. Considering that the thumb domain of GH11 xylanases defines the geometry and width of the substrate-binding cleft [24,26,42,43], the thumb domain of TsaGH11 is likely to undergo dynamic motions near the optimal temperature that facilitate substrate access and catalysis.

Meanwhile, because these simulations were performed in the absence of substrate and no experimental validation of thumb-domain function was carried out, the proposed functional role of the thumb domain should be regarded as a hypothesis based on the observed conformational dynamics. Further studies, including substrate-bound structural analyses and biochemical experiments, will be required to validate the functional significance of these conformational changes. Meanwhile, in the present study, only a single MD simulation was performed for TsaGH11 because of computational resource limitations. Therefore, additional MD simulations with multiple replicates and over a broader temperature range will be required in future studies to improve the statistical robustness of the results.

**Figure 7:**
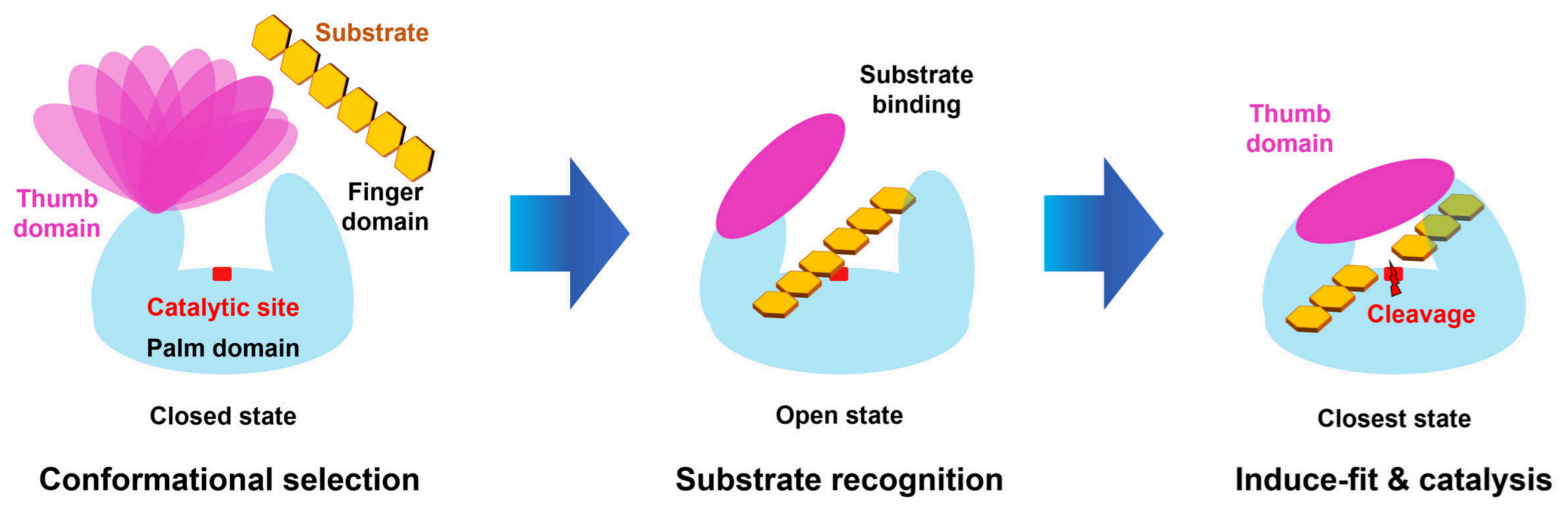
Proposed substrate recognition mechanism of TsaGH11 at its optimal enzymatic temperature.

Based on the MD simulation results and the literature, a hypothetical substrate recognition mechanism for TsaGH11 at its optimal enzymatic temperature is proposed (Figure 7): (1) At the optimal temperature, TsaGH11 is proposed to predominantly adopt a closed substrate-binding cleft formed by the thumb and finger domains, while the thumb domain dynamically samples a wide range of open and closed conformations, which may increase the probability of substrate capture. (2) Upon substrate access to the substrate-binding cleft, the cleft may transiently adopt a more open conformation accompanied by rearrangements in the distances between catalytic residues. (3) Subsequently, similar to an induced-fit mechanism, the thumb and finger domains may shift toward a closed conformation, thereby facilitating the catalytic reaction. Collectively, these observations suggest that TsaGH11 may utilize a conformational selection-like mechanism during substrate recognition. Upon substrate binding, TsaGH11 may utilize an induced-fit-like process to achieve efficient catalysis at the optimal temperature.

In this study, the proposed mechanism of substrate recognition is based on the conformational dynamics of the apo-state TsaGH11 observed during MD simulations. To validate the proposed mechanism and better understand substrate recognition, future studies involving substrate-bound crystal structures, ligand-binding assays, and complementary computational approaches will be necessary.

Analyses of RMSD, Rg, and SASA values as well as secondary structures indicated that the overall β-jelly roll fold of TsaGH11 was preserved at 300–340 K. However, local thermal unfolding of the β-roll core was observed at 360 K. Enzyme activity assays using beechwood xylan have shown that TsaGH11 reached maximal activity at 70 °C; activity decreased to approximately 40% and 30% at 80 °C and 90 °C, respectively [28]. MD simulation results suggest that the loss of enzymatic activity at high temperatures may be caused by the local thermal unfolding and structural destabilization of TsaGH11. MD simulations at increased temperatures can identify flexible or temperature-sensitive regions in proteins that are suitable targets for thermostability engineering by revealing conformational flexibility and unfolding hotspots that correlate with reduced thermal resistance [44,45,46]. In this study, thermal unfolding of the β5 and β11 strand regions was identified in both TsaGH11^closed^ and TsaGH11^open^ at 360 K. Because these thermally sensitive regions are on the opposite side of the active site and are not directly involved in substrate recognition or catalytic reactions, they represent promising targets for rational protein engineering aimed at enhancing the thermostability of TsaGH11.

## 4. Materials and Methods

### 4.1. Protein Preparation and Crystallization

Protein expression, purification, and crystallization were performed as described [28]. Briefly, recombinant DNA containing the TsaGH11 gene (UniProt code: I3VTR8) was transformed into *Escherichia coli* BL21(DE3). The cells were cultured in LB broth supplemented with kanamycin (100 μg/mL), and protein expression was induced with 0.5 mM IPTG at 18 °C overnight. Cells were harvested and disrupted by sonication, and cell debris was removed by centrifugation at 14,000 rpm for 1 h. The supernatant was purified using a Ni–NTA affinity column followed by size-exclusion chromatography on a Sephacryl S-100 column (Cytiva, Uppsala, Sweden) equilibrated with buffer containing 10 mM Tris–HCl (pH 8.0) and 200 mM NaCl. Protein purity was monitored by SDS–PAGE, and protein concentration was determined using the Bradford assay. The purified protein was concentrated using a Centricon concentrator (Merck, Burlington, MA, USA; molecular weight cutoff 10 kDa). Crystallization was performed using the hanging-drop vapor diffusion method at 22 °C. The TsaGH11 solution (~30 mg/mL, 2 μL) was mixed with an equal volume of crystallization solution containing 0.1 M sodium acetate (pH 4.6) and 3.5–4.0 M ammonium acetate. The drops were equilibrated against 500 μL of reservoir solution in 24-well VDX plates. TsaGH11 crystals appeared within 2 weeks.

### 4.2. Data Collection

X-ray diffraction data were collected at beamline 11C of the Pohang Accelerator Laboratory (PLS-II, Pohang, Republic of Korea) [47]. TsaGH11 crystals were soaked for 5 s in a cryoprotectant solution consisting of the crystallization solution supplemented with 20% (*v*/*v*) ethylene glycol. Diffraction data were collected at 100 K and recorded using a Pilatus 6M detector (Dectris, Baden-Dättwil, Switzerland). Diffraction data were processed using the Xia2 program (version 0.3.8.0) [48] at the Global Science Data hub Center (GSDC), Korea Institute of Science and Technology Information (KISTI, Daejeon, Republic of Korea) [49].

### 4.3. Structure Determination

The phase problem was solved by molecular replacement using MOLREP [50] implemented in the CCP4 suite (version 8.0.012) [51]. The crystal structure of TsaGH11 (PDB code: 8YYN) [29] was used as the search model. Model building and structure refinement were performed using COOT (version 0.9.8.7) [52] and phenix.refine in the PHENIX (version 1.20.1-4487) [53] software package, respectively. The structure of the final model was validated using MolProbity (version 4.5.1) [54].

### 4.4. MD Simulation

The crystal structures of the open and closed conformations of TsaGH11 determined in this study were used as the initial model structures for MD simulations. All-atom MD simulations were performed using GROMACS (version 2024.4) [55]. The AMBER99SB-ILDN [56] force field was applied, and the system was solvated in a cubic box with TIP3P [57] water molecules, ensuring a minimum distance of 1 nm between the protein surface and the box boundary. Counterions were added to neutralize system charge, and energy minimization was performed using the steepest descent algorithm. Long-range electrostatic interaction calculations used the Particle Mesh Ewald (PME) [58] method. NVT equilibration was performed at the target temperature for 100 ps using the V-rescale thermostat [59], with bond constraints applied using the LINCS [60] algorithm. NPT equilibration was performed for 100 ps at the target temperature using the V-rescale thermostat and at 1 bar using the Parrinello–Rahman barostat [61], with bond constraints applied using the LINCS algorithm. Production MD simulations were performed at 300, 320, 340, and 360 K for 500 ns, and the time step of MD simulations was 2 fs.

### 4.5. MD Data Analysis

Trajectory analyses, including root-mean-square deviation (RMSD), root-mean-square fluctuation (RMSF), radius of gyration (Rg), and solvent-accessible surface area (SASA), were performed using built-in GROMACS analysis tools. The representative structure was obtained by RMSD-based clustering with a cutoff of 2 Å. The centroid of the most populated cluster was selected from the equilibrated trajectories after 20 ns. Principal component analysis (PCA) and free energy landscape (FEL) analyses were performed using GROMACS based on the first two principal components (PC1 and PC2). Structural figures were generated using PyMOL (The PyMOL Molecular Graphics System, Version 2.5, Schrödinger, LLC, New York, NY, USA).

## 5. Conclusions

In this study, MD simulations of TsaGH11 were performed at various temperatures to investigate its substrate recognition mechanism. MD analyses revealed that TsaGH11 preferentially adopts a closed conformation of the substrate-binding cleft at the optimal enzymatic temperature, whereas the thumb domain exhibits increased fluctuations. Based on the MD results and the literature, it is proposed that substrate recognition involves a conformational selection-like process followed by an induced-fit-like mechanism. Furthermore, the identification of thermally sensitive regions on the opposite side of the substrate-binding cleft provides useful insights for enzyme engineering aimed at enhancing catalytic activity while improving thermostability. These results deepen our understanding of the molecular mechanism of TsaGH11 catalysis and expand the current knowledge of GH11 xylanases in industrial applications.

## Figures and Tables

**Table 1:** Data collection and refinement statistics.

Data Collection	TsaGH11
X-ray source	Beamline 11C, PLS-II
Space group	P4_1_2_1_2
Cell dimension	
a, b, c (Å)	73.37, 73.37, 165.92
α, β, γ (°)	90.00, 90.00, 90.00
Resolution (Å)	73.37–1.40 (1.42–1.40)
Unique reflections	89,334 (3947)
Completeness (%)	99.4 (90.4)
Multiplicity	21.2 (7.8)
I/sigma	11.6 (1.00)
CC1/2	0.997 (0.515)
Refinement	
Resolution (Å)	67.11–1.40
R_work_ ^a^	0.1748
R_free_ ^b^	0.1884
RMS deviations	
Bonds (Å)	0.014
Angles (°)	1.237
*B* factors (Å^2^)	
Protein	18.62
Water	35.20
Ramachandran plot	
Favored (%)	98.07
Allowed (%)	1.93
Disallowed (%)	0.00
PDB code	23AC

Values for the outer shell are given in parentheses. ^a^ R_work_ = Σ||F_obs_| − Σ|F_calc_||/Σ|F_obs_|, where Fobs and Fcalc are the observed and calculated structure factor amplitudes, respectively. ^b^ R_free_ was calculated as R_work_ using a randomly selected subset (5%) of unique reflections not used for structural refinement.

**Table 2:** Population of open and closed conformations of TsaGH11 during MD simulations at different temperatures.

Temperature	Open Conformation (%)	Closed Conformation (%)
300 K	82.69	17.31
320 K	94.65	5.35
340 K	59.00	41.00
360 K	91.19	8.81

Open and closed conformations were classified based on the Trp36–Pro143 distance using a cutoff value of 5 Å.

## Data Availability

The structure factor and coordinates were deposited at the Protein Data Bank (https://www.rcsb.org) under access code 23AC. The data that support the findings of this study are available upon request from the corresponding author.

## Supplementary Materials

The following supporting information can be downloaded at https://www.mdpi.com/article/10.3390/ijms27156869/s1.
