# Supplementary material for: Temperature-Dependent Thumb Domain Dynamics of Xylanase TsaGH11: Insights from Molecular Dynamics Simulations
Source: Int J Mol Sci. 2026 Jul 31;27(15):6869. doi: 10.3390/ijms27156869 (PMC13466707; doi:10.3390/ijms27156869)
Supplement: Supplementary file 1 [file ijms-27-06869-s001.zip › ijms-4417487-supplementary.pdf]

Supplementary Data

Temperature-dependent thumb domain dynamics of xylanase TsaGH11 revealed by molecular dynamics simulations

Ki Hyun Nam*

College of General Education, Kookmin University, Seoul 02707, Republic of Korea

*Corresponding: structure@kookmin.ac.kr

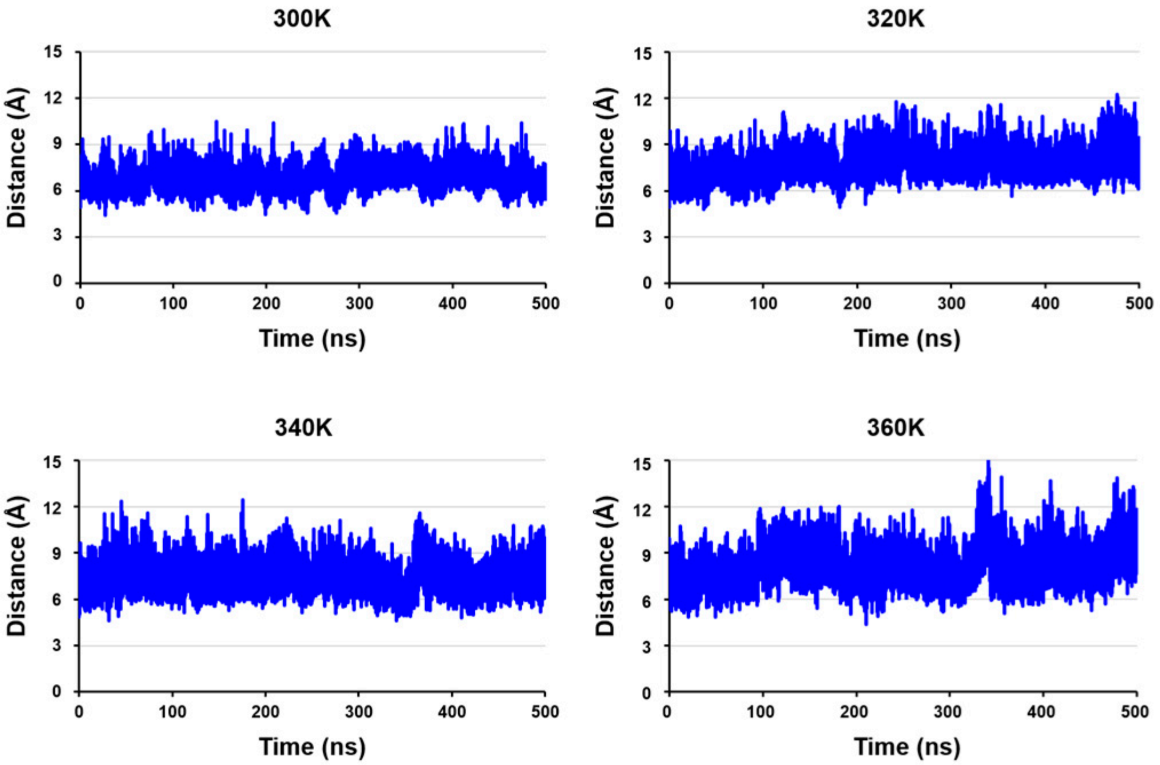

Supplementary Figure S1. Time-series analysis of the Glu105–Glu198 distance during MD simulations of TsaGH11 at 300, 320, 340, and 360 K.

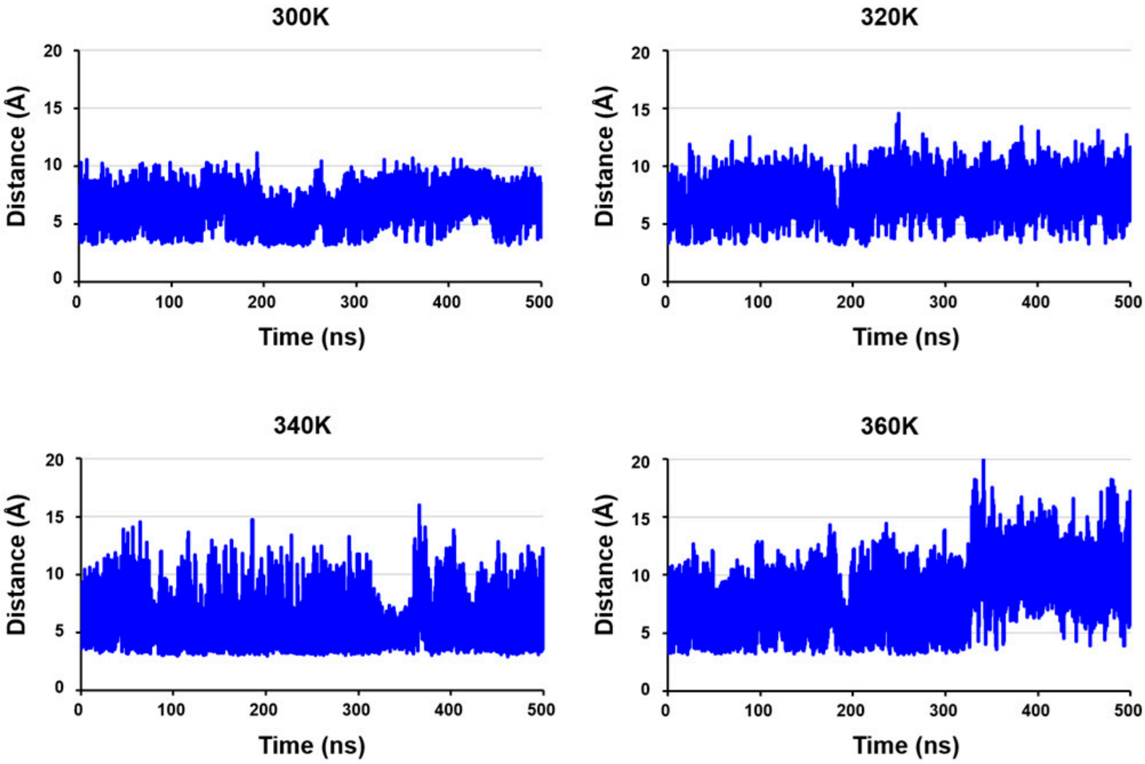

Supplementary Figure S2. Time-series analysis of the Trp36–Pro143 distance during MD simulations of TsaGH11 at 300, 320, 340, and 360 K.

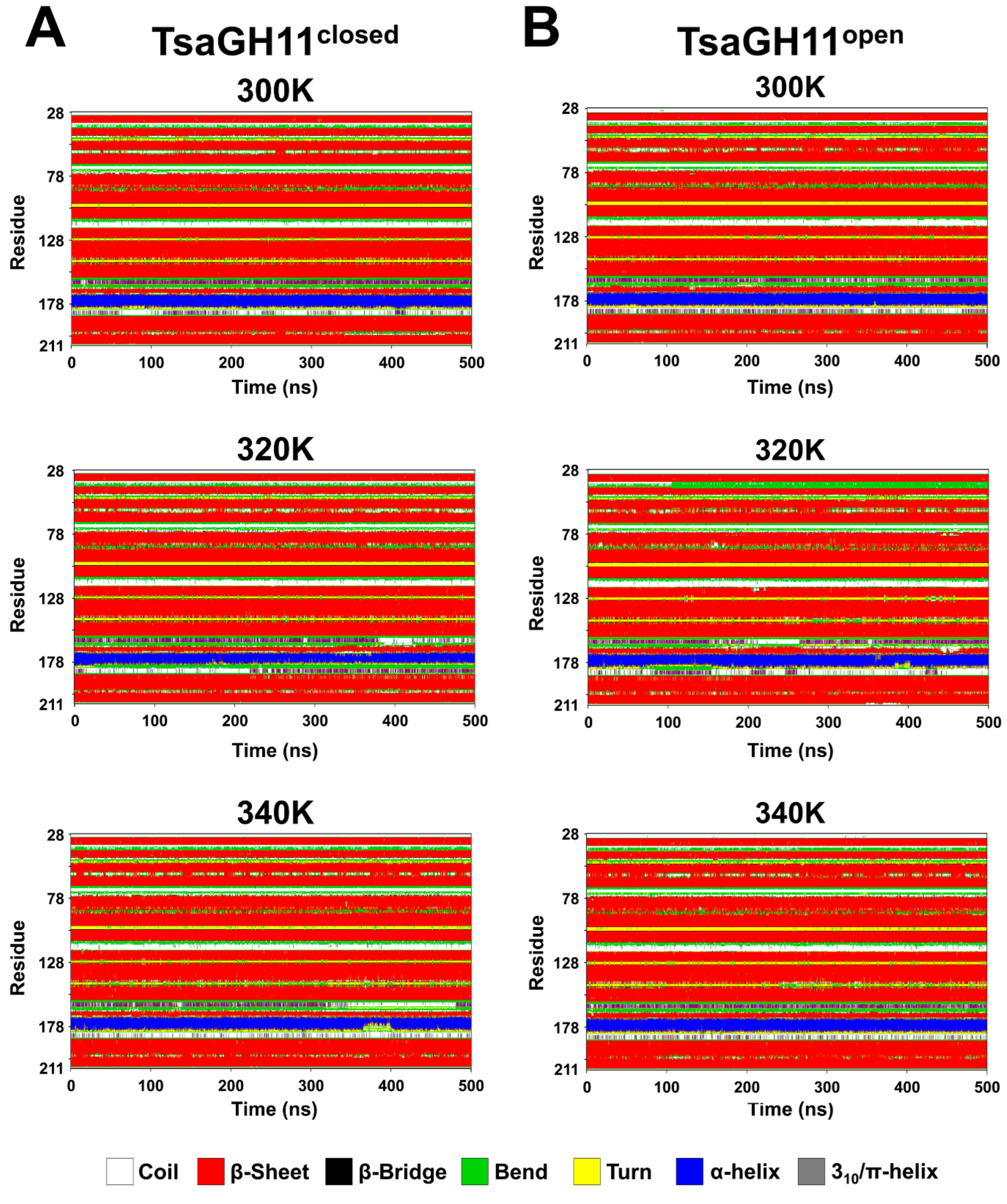

Supplementary Figure S3. Time evolution of the secondary structures of (A) *Tsa*GH11^{close} and (B) *Tsa*GH11^{open} at 300, 320, and 360 K over the 500 ns molecular dynamics production trajectories.
